# Botulism due to Injection Drug Use

**DOI:** 10.21980/J8Q93B

**Published:** 2023-04-30

**Authors:** Timothy Hoffman, Jennifer Yee

**Affiliations:** * The Ohio State University, Department of Emergency Medicine, Columbus, OH

## Abstract

**Audience:**

This scenario was developed to educate emergency medicine residents on the diagnosis and management of wound botulism secondary to injection drug use.

**Introduction:**

Botulism is a relatively rare cause of respiratory failure and descending weakness in the United States, caused by prevention of presynaptic acetylcholine release at the neuromuscular junction. This presentation has several mimics, including myasthenia gravis and the Miller-Fisher variant of Guillain-Barré. It may be caused by ingestion of spores (infant), ingestion of pre-formed toxin (food-borne), formation of toxin *in vivo* (wound-associated cases), through weaponized sources, or through inappropriately administered injections (iatrogenic). Cases of black tar heroin injection have been associated with botulism. Regardless of the etiology, prompt assessment and support of respiratory muscle strength and ordering antidotal therapy is key to halting further muscle weakness progression.

**Educational Objectives:**

At the conclusion of the simulation session, learners will be able to: 1) Identify the different etiologies of botulism, including wound, food-borne, infant, iatrogenic, and inhalational sources, 2) describe the pathophysiology of botulism toxicity and how it prevents presynaptic acetylcholine release at the neuromuscular junction, 3) develop a differential for bilateral descending muscle weakness, 4) compare and contrast presentations of myasthenia gravis, botulism, and the Miller-Fisher variant of Guillain-Barré syndrome, 5) describe measurement of neurologic respiratory parameter testing, such as negative inspiratory force, 6) outline treatment principles of wound-associated botulism, including antitoxin administration, wound debridement, tetanus vaccination, and evaluation for the need of antibiotics, and 7) identify appropriate disposition of the patient to the medical intensive care unit (ICU).

**Educational Methods:**

This session was conducted using high-fidelity simulation, followed by a debriefing session and lecture on the diagnosis, differential diagnosis, and management of botulism secondary to injection drug use. Debriefing methods may be left to the discretion of participants, but the authors have utilized advocacy-inquiry techniques. This scenario may also be run as an oral board case.

**Research Methods:**

Our residents are provided a survey at the completion of the debriefing session so they may rate different aspects of the simulation, as well as provide qualitative feedback on the scenario.

**Results:**

Sixteen learners completed a feedback form. This session received all six and seven scores (consistently effective/very good and extremely effective/outstanding, respectively) other than three isolated five scores. The form also includes an area for general feedback about the case at the end. Illustrative examples of feedback include: “Really awesome debrief, breakdown of pathophysiology and clinical applications. Great work!”; “Great case with awesome learning points,” and “Loved this session. Rare case but very great learning.” Specific scores are available upon request.

**Discussion:**

This is a cost-effective method for reviewing botulism diagnosis and management. The case may be modified for appropriate audiences, such as using classic illness scripting (eg, ingestion of canned foods). We encourage readers to utilize a standardized patient to demonstrate extraocular muscle weakness and bulbar symptoms to increase psychological buy-in.

**Topics:**

Medical simulation, botulism, toxicologic emergencies, toxicology, neurology, emergency medicine.

## USER GUIDE

**Table t1-jetem-8-2-s62:** 

**List of Resources:**	
Abstract	62
User Guide	64
Instructor Materials	66
Operator Materials	79
Debriefing and Evaluation Pearls	81
Simulation Assessment	83

[Table t1-jetem-8-2-s62] **Learner Audience:**

Interns, Junior Residents, Senior Residents

**Time Required for Implementation:**

**Instructor Preparation:** 30 minutes

**Time for case:** 20 minutes

**Time for debriefing:** 40 minutes

**Recommended Number of Learners per Instructor:**

3–4

**Topics:**

Medical simulation, botulism, toxicologic emergencies, toxicology, neurology, emergency medicine.

**Objectives:**

By the end of this simulation session, learners will be able to:

1. Identify the different etiologies of botulism (wound, food-borne, infant, iatrogenic, inhalational)
2. Describe the pathophysiology of botulism toxicity
3. Develop a differential for bilateral descending muscle weakness
4. Compare and contrast presentations of myasthenia gravis, botulism, and the Miller-Fisher variant of Guillain-Barré syndrome
5. Describe measurement of neurologic respiratory parameter testing
6. Outline treatment principles of wound-associated botulism, including antitoxin administration, wound debridement, tetanus vaccination, and evaluation for the need of antibiotics
7. Identify appropriate disposition of the patient to the medical intensive care unit (MICU).

### Linked objectives and methods

Patients with botulism require prompt recognition and treatment with antitoxin in order to slow progression to respiratory failure. After this scenario, providers will be able to describe the different causes of botulism toxicity (objective 1), the pathophysiology of botulism toxicity (objective 2) and compare and contrast different neuromuscular weakness pathologies that may present similarly (objectives 3 and 4). Because a neuromuscular cause of descending weakness is suspected, neurologic respiratory parameters such as a negative inspiratory force and forced vital capacity should be obtained to assess the current trajectory of respiratory failure (objective 5). Once botulism is established as a potential cause of weakness, the process of ordering antitoxin should be arranged, as well as ancillary care such as wound debridement, tetanus vaccination, and assessing need for antibiotics (objective 6) before being admitted in the medical ICU for further respiratory monitoring and management (objective 7). Objectives were tracked by facilitators taking notes during the simulation scenario for the subsequent debriefing discussion.

This simulation scenario allows learners to reinforce botulism diagnostic and management skills in a psychologically-safe learning environment, and then receive formative feedback on their performance.

### Recommended pre-reading for instructor

We recommend that instructors review literature regarding botulism, including presenting signs/symptoms, diagnosis, and management. Suggested readings include materials listed under the “References/suggestions for further reading” section below.

### Results and tips for successful implementation

This simulation was written to be performed as a high-fidelity simulation scenario, but also may be used as a mock oral board case.

The case was written for emergency medicine residents within the setting of an emergency department (ED) with a stroke center designation; however, neurology will be unavailable for consultation if called.

Starting the case off with the nurse stating that the patient was a stroke alert allowed for residents to work through triage cueing and momentum bias. Depending on the experience of the learners, this may be left in or omitted at the facilitator’s discretion.

Botulism due to injection drug use was chosen due to the uncommon presentation of wound botulism. Ultimately, only one group out of nine voiced the diagnosis of botulism during the scenario, but no one ordered the antitoxin. About half of the groups voiced a possible differential diagnosis of myasthenia gravis. During debriefing, residents gave positive feedback regarding the review of neuromuscular disorders with comparing and contrasting their clinical presentations. Facilitators may choose to use a classic illness script such as eating homemade canned foods or an infant who became constipated and weak after being fed unpasteurized honey.

Our scenario began with the patient demonstrating an earlier disease course with extraocular muscle and bulbar weakness rather than dyspnea, but then progressed to descending paralysis and early respiratory muscle weakness. We did not want to focus on respiratory failure in neuromuscular disease and how this may affect paralytic dosing; however, facilitators may elect to edit the case to emphasize these points.

We found that putting up a picture of ptosis and mydriasis emphasized that in the confederate’s ptosis exam, but it was still difficult to appreciate mydriasis on the picture. Subsequently, we had the confederate patient state, “I don’t know why my pupils are so large,” when his eyes were examined.

One of our clinical respiratory therapists participated in the case and demonstrated how to obtain respiratory parameters (negative inspiratory force, vital capacity) when the learners requested these, which was well-received.

Our residents are provided a survey at the completion of the debriefing session so they may rate different aspects of the simulation, as well as provide qualitative feedback on the scenario. The local institution’s simulation center’s electronic feedback form is based on the Center of Medical Simulation’s Debriefing Assessment for Simulation in Healthcare (DASH) Student Version Short Form^1^ with the inclusion of required qualitative feedback if an element was scored less than a six or seven.

Sixteen learners completed a feedback form. This session received all six and seven scores (consistently effective/very good and extremely effective/outstanding, respectively) other than three isolated five scores. The lowest average score was tied at 6.63 for “Before the simulation, the instructor set the stage for an engaging learning experience,” and “The instructor identified what I did well or poorly - and why.” The highest average score was 6.94 for “The instructor provoked in-depth discussions that led me to reflect on my performance.” The form also includes an area at the end for general feedback about the case. Illustrative examples of feedback include: “Really awesome debrief, breakdown of pathophysiology and clinical applications. Great work!”; “Great case with awesome learning points,” and “Loved this session. Rare case but very great learning.” Specific scores are available upon request.

## Supplementary Information



## Figures and Tables

**Table t2-jetem-8-2-s62:** 

#1	Picture of a person’s face with ptosis
#2	Electrocardiogram, sinus bradycardia
#3	Chest X-ray
#4	Computed Tomography image, head
#5	Complete blood count (CBC)
#6	Basic metabolic panel (BMP)
#7	Liver function panel (LFTs)
#8	Magnesium
#9	Thyroid stimulating hormone (TSH)
#10	Lactate
#11	Troponin
#12	Arterial Blood Gas (ABG)
#13	Ethanol level
#14	Toxicology Screen
#15	Urinalysis
#16	Cerebrospinal Fluid (CSF) studies

**Table t3-jetem-8-2-s62:** 

**Vitals:** Heart Rate (HR) 60	Blood Pressure (BP) 116/86
Respiratory Rate (RR) 12	Temperature (T) 99.8°F
Oxygen Saturation (O_2_Sat) 96% on room air	

**Figure f1-jetem-8-2-s62:**
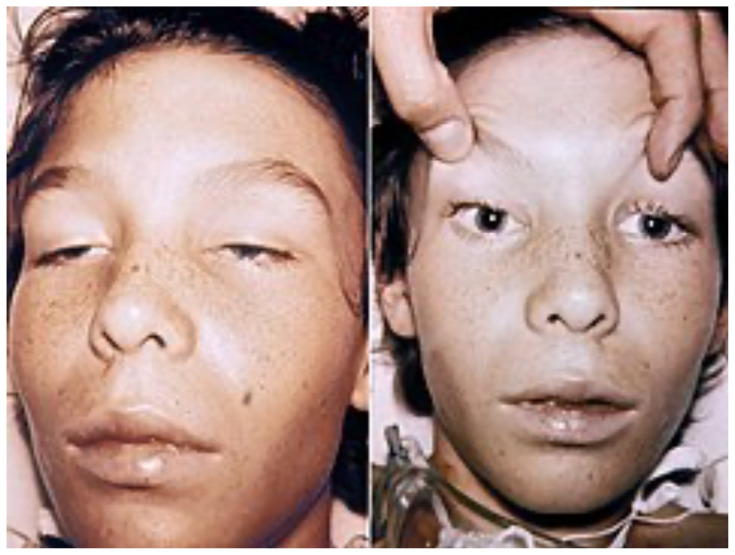


**Figure f2-jetem-8-2-s62:**
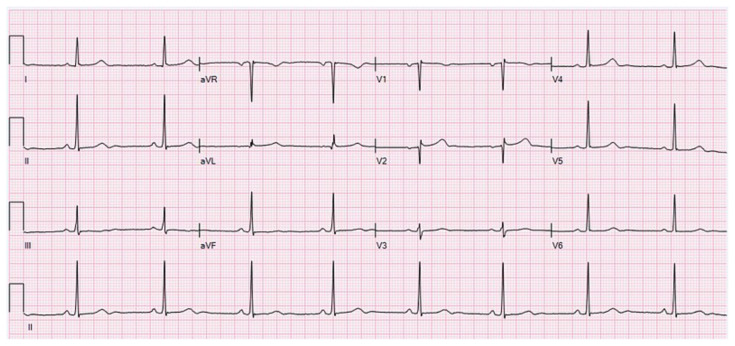


**Figure f3-jetem-8-2-s62:**
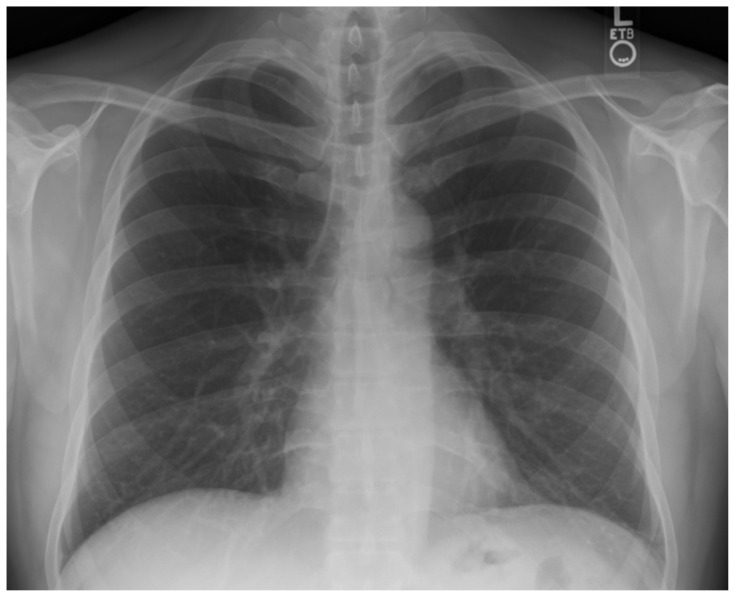


**Figure f4-jetem-8-2-s62:**
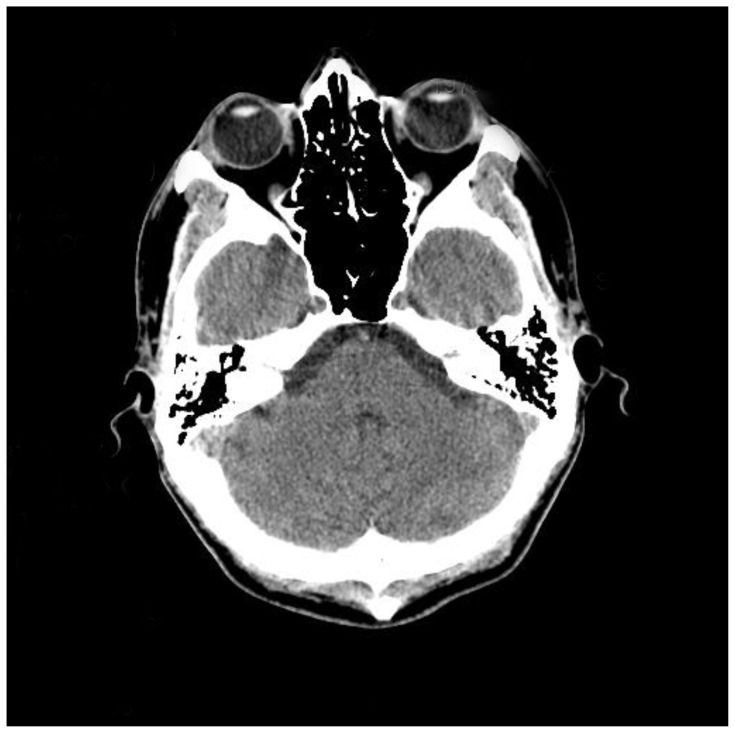


**Table t4-jetem-8-2-s62:** 

*Complete blood count (CBC)*	
White blood count (WBC)	13.8 × 1000/mm^3^
Hemoglobin (Hgb)	13.8 g/dL
Hematocrit (HCT)	28.4%
Platelet (Plt)	450 × 1000/mm^3^
Bands	17%
*Basic metabolic panel (BMP)*	
Sodium	143 mEq/L
Potassium	4.1 mEq/L
Chloride	98 mEq/L
Bicarbonate (HCO_3_)	24 mEq/L
Blood Urea Nitrogen (BUN)	35 mg/dL
Creatinine (Cr)	1.0 mg/dL
Glucose	88 mg/dL
Calcium	8.1 mg/dL
*Liver function test (LFTs)*	
Total bilirubin	0.7 mg/dL
Direct bilirubin	0.3 mg/dL
Alkaline phosphatase	97 units/L
Aspartate aminotransferase (AST)	31 units/L
Alanine aminotransferase (ALT)	42 units/L
Albumin	2.7 g/dL
*Magnesium*	1.9 mEq/L
*Thyroid stimulating hormone (TSH)*	3.64 ulU/mL (0.550–4.780 ulU/mL)
*Lactate*	1.5 mmol/L (<1.0 mmol/L)
*Troponin*	<0.015 ml/mL
*Arterial Blood Gas (ABG)*	
pH	7.31
pCO_2_	50 mmHg
pO_2_	86 mmHg
HCO_3_	23 mEq/L
O_2_ Saturation	96% on room air
*Ethanol level*	<0.01 mg/dL
*Toxicology Screen*	
Amphetamines	Negative
Barbiturates	Negative
Benzodiazepines	Negative
Cocaine	Negative
Methadone	Negative
Opiates	Positive
Oxycodone	Negative
PCP	Negative
THC	Positive
*Urinalysis*	
Leukocyte esterase	Negative
Nitrites	Negative
Blood	None
Protein	None
Ketones	1+
Glucose	None
Color Dark	Yellow
White blood cells	0–5 WBCs/HPF
Red blood cells	0–5 RBCs/HPF
Squamous epithelial cells	5–10 cells/HPF
Specific gravity	1.015
*Cerebrospinal Fluid (CSF) studies*	
Appearance	Clear
Glucose	60 mg/dL
Protein	40 mg/dL
White blood cells	0–5 RBCs/uL
Red blood cells	0–3 WBCs/uL
Gram stain	No organisms seen

**Table t5-jetem-8-2-s62:** SIMULATION EVENTS TABLE:

Minute (state)	Participant action/trigger	Patient status (simulator response) & operator prompts	Monitor display (vital signs)
0:00 (Baseline)	SP nurse brings the team into the patient’s room in the emergency department.	Participants should begin by placing the patient on a monitor, obtaining history from patient, obtaining a point-of-care glucose, and performing a physical exam. If asked to go to CT before a neurologic physical exam has been performed, the CT scanner is currently busy with trauma patients.	A: T 99.8°F HR 60 BP 116/86 RR 12 O_2_ sat 96% room air (RA)
6:00	Team should recognize the bilateral ptosis, mydriasis, and dysarthria. IV placed, labs obtained. Labs, EKG should be ordered.	CT will be available to the learners once a neurologic exam has been performed. If the team recognizes botulism and orders antitoxin by minute 6, vitals remain unchanged from vitals (B). If the team does not order the antitoxin by minute 6, bradycardia worsens and patient develops dyspnea/hypoxia (C). The patient can also say, “Hey, I can’t shrug my shoulders,” and give a weak/minimal shrug to demonstrate descending muscle weakness. If team doesn’t order a CT scan, nursing will remind them that a stroke alert was called and ask them if they still want to go to the CT scanner. If neurology is called before CT head is interpreted by the team, the nurse will tell the participants that the neurologist is busy and therefore unavailable for consultation at this time. If neurology is called after CT head is interpreted by the team, neurology will remark that the patient does not sound like a tPA candidate and leave the conversation abruptly	B: T 99.8°F HR 48 BP 116/86 RR 12 O_2_ sat 94% RA
8:00 (Case Completion)	Team should recognize worsening dyspnea and hypoxia. Should order neurologic respiratory parameters and order the antitoxin	If team orders respiratory parameters and antitoxin, vitals remain unchanged (C). If respiratory parameters are not ordered by minute 12, patient becomes more dyspneic with shallow breathing (D). If antidote is not ordered by minute 12, vitals worsen more markedly (E). Case ends after antidote is ordered, respiratory parameters obtained, and the MICU is contacted for admission (or minute 15/facilitator’s discretion). If the team does not ask to call for admission, the nurse should prompt, “We need this bed; triage is full. What are we doing for dispo?”	C: T 99.8°F HR 40 BP 116/86 RR 12 O_2_ sat 88% RA O_2_ sat 92% on NRB or nasal cannula oxygen D: T 99.8°F HR 38 BP 116/86 RR 26 O_2_ sat 85% RA O_2_ sat 88% on NRB or nasal cannula oxygen E: T 99.8°F HR 35 BP 116/86 RR 30 O_2_ sat 85% RA O_2_ sat 88% on NRB or nasal cannula oxygen

**Table t6-jetem-8-2-s62:** 

Obtain an early (within first 2 minutes) point-of-care glucose Perform a full neurologic exam, including reflexes Fully expose patient to reveal antecubital track marks Contact poison control or the health department to order antitoxin Order neurologic respiratory parameters 6. Admit to the medical ICU	0:00

**Table t7-jetem-8-2-s62:** 

	Milestone	Did not achieve level 1	Level 1	Level 2	Level 3
1	**Emergency Stabilization (PC1)**	□ Did not achieve Level 1	□ Recognizes abnormal vital signs	□ Recognizes an unstable patient, requiring intervention Performs primary assessment Discerns data to formulate a diagnostic impression/plan	□ Manages and prioritizes critical actions in a critically ill patient Reassesses after implementing a stabilizing intervention
2	**Performance of focused history and physical (PC2)**	□ Did not achieve Level 1	□ Performs a reliable, comprehensive history and physical exam	□ Performs and communicates a focused history and physical exam based on chief complaint and urgent issues	□ Prioritizes essential components of history and physical exam given dynamic circumstances
3	**Diagnostic studies (PC3)**	□ Did not achieve Level 1	□ Determines the necessity of diagnostic studies	□ Orders appropriate diagnostic studies. Performs appropriate bedside diagnostic studies/procedures	□ Prioritizes essential testing Interprets results of diagnostic studies Reviews risks, benefits, contraindications, and alternatives to a diagnostic study or procedure
4	**Diagnosis (PC4)**	□ Did not achieve Level 1	□ Considers a list of potential diagnoses	□ Considers an appropriate list of potential diagnosis Considers an appropriate list of potential diagnosis	□ Makes the appropriate diagnosis Considers other potential diagnoses, avoiding premature closure
5	**Pharmacotherapy (PC5)**	□ Did not achieve Level 1	□ Asks patient for drug allergies	□ Selects an medication for therapeutic intervention, consider potential adverse effects	□ Selects the most appropriate medication and understands mechanism of action, effect, and potential side effects Considers and recognizes drug-drug interactions
6	**Observation and reassessment (PC6)**	□ Did not achieve Level 1	□ Reevaluates patient at least one time during case	□ Reevaluates patient after most therapeutic interventions	□ Consistently evaluates the effectiveness of therapies at appropriate intervals
7	**Disposition (PC7)**	□ Did not achieve Level 1	□ Appropriately selects whether to admit or discharge the patient	□ Appropriately selects whether to admit or discharge Involves the expertise of some of the appropriate specialists	□ Educates the patient appropriately about their disposition Assigns patient to an appropriate level of care (ICU/Tele/Floor) Involves expertise of all appropriate specialists
9	**General Approach to Procedures (PC9)**	□ Did not achieve Level 1	□ Identifies pertinent anatomy and physiology for a procedure Uses appropriate Universal Precautions	□ Obtains informed consent Knows indications, contraindications, anatomic landmarks, equipment, anesthetic and procedural technique, and potential complications for common ED procedures	□ Determines a back-up strategy if initial attempts are unsuccessful Correctly interprets results of diagnostic procedure
20	**Professional Values (PROF1)**	□ Did not achieve Level 1	□ Demonstrates caring, honest behavior	□ Exhibits compassion, respect, sensitivity and responsiveness	□ Develops alternative care plans when patients’ personal beliefs and decisions preclude standard care
22	□ **Patient centered communication (ICS1)**	□ Did not achieve level 1	□ Establishes rapport and demonstrates empathy to patient (and family) Listens effectively	□ Elicits patient’s reason for seeking health care	□ Manages patient expectations in a manner that minimizes potential for stress, conflict, and misunderstanding. Effectively communicates with vulnerable populations, (at risk patients and families)
23	**Team management (ICS2)**	□ Did not achieve level 1	□ Recognizes other members of the patient care team during case (nurse, techs)	□ Communicates pertinent information to other healthcare colleagues	□ Communicates a clear, succinct, and appropriate handoff with specialists and other colleagues Communicates effectively with ancillary staff

## INSTRUCTOR MATERIALS

**Case Title:** Botulism due to Injection Drug Use

**Case Description & Diagnosis (short synopsis):** Patient is a 37-year-old male who presents to triage by private vehicle with two hours of blurry vision. Upon further questioning in triage, he disclosed four days of difficulty speaking. Triage called a stroke alert and requested an immediate evaluation prior to going to the computed tomography (CT) scanner. If asked to go to CT before a neurologic physical exam has been performed, the CT scanner is currently busy with trauma patients.

Participants should request a point-of-care glucose and perform a full neurologic exam on the patient, which should reveal symmetric ptosis and slurred speech. Exposure of the patient’s arms reveal bilateral track marks with surrounding erythema. When directly questioned, patient discloses injection drug use. At this point, participants should suspect other differentials such as myasthenia gravis or botulism. The patient will experience worsening bradycardia and develop dyspnea with delays in diagnosis or initiation of treatment; however, respiratory parameters should be reassuring against immediate need for intubation. Poison control or the health department should be contacted to obtain antivenom to treat for botulism. The patient should ultimately be dispositioned to the medical intensive care unit.

**Equipment or Props Needed:**

- High fidelity simulation mannequin (authors used a SimMan® 3G Manikin) with lumbar puncture task trainer (eg: Kyoto Kagaku Lumbar Puncture Simulator IIa) and lumbar puncture kit kept under a bedsheet
- Angiocatheters for peripheral intravenous access = 18g, 20g, 22g
- Cardiac monitor
- Pulse oximetry
- IV pole
- Bilevel positive airway pressure (BiPAP) or continuous positive airway pressure (CPAP) mask
- Non-rebreather mask
- Nasal cannula
- Wound moulage to mock up drug injection skin tracts
- Long button-up shirt for the confederate patient to wear
- Normal saline (1 liter ×2)
- Lactated Ringer’s (1 liter ×2)
- Simulated medications with labeling: cefepime, piperacillin-tazobactam, vancomycin

**Confederates needed:**

*One confederate as the primary nurse and one confederate as the patient. Faculty may call in overhead as the neurology, department of health, or poison control center provider*

**Stimulus Inventory:** [Table t2-jetem-8-2-s62]

**Background and brief information:** Patient is a 37-year-old male who presents to your ED via triage for blurry vision for the past two hours. A stroke alert was called and you were asked to come assess him immediately.

**Initial presentation:** 37-year-old male lying supine on the cot, eyes half open, conversant

- Past medical history: injection heroin use (only disclosed if asked directly about illicit drug use)
- Past surgical history: none
- Medications: none
- Allergies: none
  Social hx: smokes cigarettes. Denies alcohol use. Injection heroin use (does not disclose this or marijuana use unless asked specifically about illicit drug use.)
- Family history: noncontributory
- Vital signs:
  ○ HR – 60 beats per minute
  ○ Resp rate – 12 respirations per minute
  ○ Temp – 99.8° F
  ○ BP – 116/86 mmHg
  ○ Pulse ox – 96% on room air
- Weight: 90 kg

Assessment: Lying supine, conversational

**How the scene unfolds:** Patient is a 37-year-old male who presents to triage by private vehicle with two hours of blurry vision. Upon further questioning in triage, he disclosed four days of difficulty speaking. Triage called a stroke alert and requested an immediate evaluation prior to going to the CT scanner. If asked to go to CT before a neurologic physical exam has been performed, the CT scanner is currently busy with trauma patients.

Patient’s vitals are initially stable. Participants should request a point-of-care glucose and perform a full neurologic exam on the patient, played by a confederate actor, which should reveal symmetric ptosis and slurred speech. When the eyes specifically are examined, participants are shown a picture of a patient with ptosis and mydriasis. Exposure of the patient’s arms reveal bilateral track marks with surrounding erythema. When directly questioned, patient discloses injection drug use. At this point, participants should suspect other differentials such as myasthenia gravis or botulism. The patient will experience worsening bradycardia and develop dyspnea with mild hypoxia with delays in diagnosis or initiation of treatment at minutes six and twelve; however, respiratory parameters should be reassuring against immediate need for intubation, and patient will still be able to speak in complete sentences without somnolence.

Participants may obtain a CT scan of the head or perform a lumbar puncture to evaluate for the albuminocytologic dissociation seen with myasthenia gravis; however, labs and imaging will be largely unrevealing. Neurology will be initially unavailable for consultation, but if called after CT head is interpreted, they will remark that the patient does not sound like a tissue plasminogen activator (tPA) candidate and leave the conversation abruptly. Poison control or the health department should be contacted to obtain antivenom to treat for botulism. The patient should ultimately be dispositioned to the medical intensive care unit.

**Critical actions:**

1. Obtain an early (within first 2 minutes) point-of-care glucose
2. Perform a full neurologic exam, including reflexes
3. Fully expose patient to reveal antecubital track marks
4. Contact poison control or the health department to order antitoxin
5. Order neurologic respiratory parameters
6. Admit to the medical ICU

**Case Title:** Botulism due to Injection Drug Use

**Chief Complaint:** Blurry vision[Table t3-jetem-8-2-s62]

**General Appearance:** Lying supine on the cot, conversational.

**Primary Survey:**

- **Airway:** patent
- **Breathing:** clear to auscultation bilaterally
- **Circulation:** heart regular rate and rhythm, 2+ symmetric pulses, capillary refill >3 seconds

**History:**

- **History of present illness:** Patient reports four days of trouble speaking and two hours of blurry vision. Denies headache, unilateral weakness or paresthesias, upper respiratory complaints, chest pain, dyspnea, cough, vomiting, diarrhea, dysuria, urinary frequency, rash, or myalgias.
  ○ If asked:
    ▪ Last tetanus immunization is unknown
    ▪ Denies recent falls
    ▪ No history of seizures
- **Past medical history**: injection heroin use and marijuana use (does not disclose unless asked specifically about illicit drug use)
- **Past surgical history:** none
- **Medications:** none
- **Allergies:** none
- **Social history:** smokes cigarettes. Denies alcohol use. Injection heroin use (does not disclose unless asked specifically about illicit drug use)
- **Family history:** noncontributory

**Secondary Survey/Physical Examination:**

- **Vital signs**:
  ○ HR – 60
  ○ R – 12
  ○ Temp – 99.8°F
  ○ BP – 116/86 right upper extremity
  ○ Pulse ox – 98% room air
  ○ Weight: 90 kg
- **General appearance:** lying supine on cot, appears stated age
- **HEENT:**
  ○ **Head:** atraumatic
  ○ **Eyes:** bilateral ptosis (picture of ptosis/fixed mydriasis is shown when team specifically examines the patient’s eyes)
  ○ **Ears:** within normal limits (wnl)
  ○ **Nose:** wnl
  ○ **Throat:** wnl
- **Neck:** wnl
- **Heart:** regular rhythm and rate. Otherwise within normal limits
- **Lungs:** wnl
- **Abdominal/GI:** wnl
- **Genitourinary:** deferred/within normal limits
- **Rectal:** deferred/within normal limits
- **Extremities:** bilateral track marks with surrounding erythema seen in antecubital fossa. Otherwise within normal limits
- **Neuro:** bilateral ptosis, fixed mydriasis, slurred speech. Otherwise within normal limits
- **Skin:** bilateral track marks with surrounding erythema seen in antecubital fossa. Otherwise within normal limits
- **Lymph:** wnl
- **Psych:** wnl

*Picture of a person’s face with ptosis*

Fred HL, van Dijk HA. Botulism 1 and 2. In: Wikimedia Commons.

https://commons.wikimedia.org/wiki/File:Botulism1and2.JPG. Public domain.[Fig f1-jetem-8-2-s62]

*Electrocardiogram, sinus bradycardia*

Ewingdo. ECG Sinus Bradycardia 49 bpm. In: Wikimedia Commons.

https://commons.wikimedia.org/wiki/File:ECG_Sinus_Bradycardia_49_bpm.jpg. Public domain.[Fig f2-jetem-8-2-s62]

*Chest X-ray*

Stillwaterising. Chest Xray PA 3-8-2010. In: Wikimedia Commons. File:Chest Xray PA 3-8-2010.png - Wikimedia Commons. Public domain.[Fig f3-jetem-8-2-s62]

*Computed Tomography image, head*

Ciscel A. Head CT scan. In: Wikimedia Commons.

https://commons.wikimedia.org/wiki/File:Head_CT_scan.jpg. Public domain.[Fig f4-jetem-8-2-s62]

[Table t4-jetem-8-2-s62]

## OPERATOR MATERIALS

[Table t5-jetem-8-2-s62] **Diagnosis:**

Botulism due to Injection Drug Use

**Disposition:**

MICU

## DEBRIEFING AND EVALUATION PEARLS

### Botulism

Categories of botulism^2^

- Infant botulism: *C. botulinum*. after the **ingestion of clostridial spores** that then colonize the host’s gastrointestinal (GI) tract and release toxin produced in vivo
  ○ honey if given to an infant <12 months of age because they do not possess colonization of intestinal flora yet
    ▪ muscle weakness may be preceded with constipation
    ▪ treatment: BabyBIG (California Department of Health)
  ○ Adult enteric infectious botulism is rare
- Food-borne botulism - after **ingestion of preformed botulinum toxin** (contaminated food)
  ○ canned goods
  ○ could also be from intentional placement in food
- Wound botulism. *C. botulinum* produces toxin *in vivo*
  ○ results from deep/contaminated wound
  ○ toxin gets absorbed into the bloodstream
  ○ soldiers, open wounds, injection drug use (often subcutaneous or intradermal)
- rare: iatrogenic
- rare: inhalational botulism from inhaling aerosolized toxin

Botulism toxin is the most potent bacterial toxin known^2^. The toxin binds presynaptic voluntary motor and autonomic neuromuscular junction cells and prevents presynaptic release of acetylcholine and subsequent postsynaptic membrane depolarization.

CDC’s key features:

- absence of fever
- (descending) symmetric neurologic deficits, usually bilateral
  ○ Blurry vision: from fixed mydriasis and CN III, IV, VI palsies
  ○ Bulbar weakness
- remains responsive
- bradycardic/normal heart rate and normal blood pressure
- lack of sensory deficits

Shortness of breath alone identified patients at increased mortality risk^4^

- Anticholinergic mydriasis, decreased salivation, urinary retention
- GI symptoms are not seen with wound botulism^3^

Differential Diagnoses include^1^:

- Myasthenia gravis: increasing fatigue with muscle use. However, pupillary function will be normal.
- Miller-Fisher variant of Guillain-Barré syndrome: ophthalmoplegia, ataxia, areflexia. May develop fixed mydriasis.

Treatment

- Assess for need for respiratory support, mechanical ventilation^1^
- Respiratory parameters that suggest weakening respiratory function (repeat each parameter several times to obtain an average):
  ○ forced vital capacity (FVC) <20 mL/kg,
  ○ maximum inspiratory pressure (ie, negative inspiratory force) <30 cm H_2_O,
    ▪ after exhaling slowly as much as possible, patients should breathe in as hard as possible, maintaining this for at least a second and a half
  ○ maximum expiratory pressure <40 cm H_2_O
- after taking as deep a breath in as possible, patients should exhale as hard as possible, maintaining this for at least a second and a half
- Antitoxin only prevents weakness progression; it does not reverse pre-existing weakness^1^
  ○ contact the state health department immediately for assistance to obtain a supply of antitoxin
- Wound associated forms of botulism: debride the wound, treat superimposed bacterial infections with antibiotics^1^, tetanus vaccination

### Closed loop communication and team interaction

- Pointed roles during a critical event are extremely important in providing maximal care
- Address team members by their roles or names when asking for an action and request a verbal confirmation that you were heard and understood

## SIMULATION ASSESSMENT

*Case: Botulism due to Injection Drug Use*

Learner: _________________________________________

***Assessment Timeline***

This timeline is to help observers assess their learners. It allows observer to make notes on when learners performed various tasks, which can help guide debriefing discussion.

**Critical Actions:** [Table t6-jetem-8-2-s62]

**Critical Actions:**

□ Obtain an early (within first 2 minutes) point-of-care glucose
□ Perform a full neurologic exam, including reflexes
□ Fully expose patient to reveal antecubital track marks
□ Contact poison control or the health department to order antitoxin
□ Order neurologic respiratory parameters
□ Admit to the medical ICU

**Summative and formative comments:**

**Milestones assessment:** [Table t7-jetem-8-2-s62]

## References

[1] Debriefing Assessment for Simulation in Healthcare (DASH) n.d Retrieved from https://harvardmedsim.org/debriefing-assessment-forsimulation-in-healthcare-dash/

