# Supplementary material for: Botulism due to Injection Drug Use
Source: J Educ Teach Emerg Med. 2023 Apr 30;8(2):S62–87. doi: 10.21980/J8Q93B (PMC10332679; doi:10.21980/J8Q93B)
Supplement: Supplementary file 1 [file JETem-8-2-S62-supp1.pptx]

## Slide 1
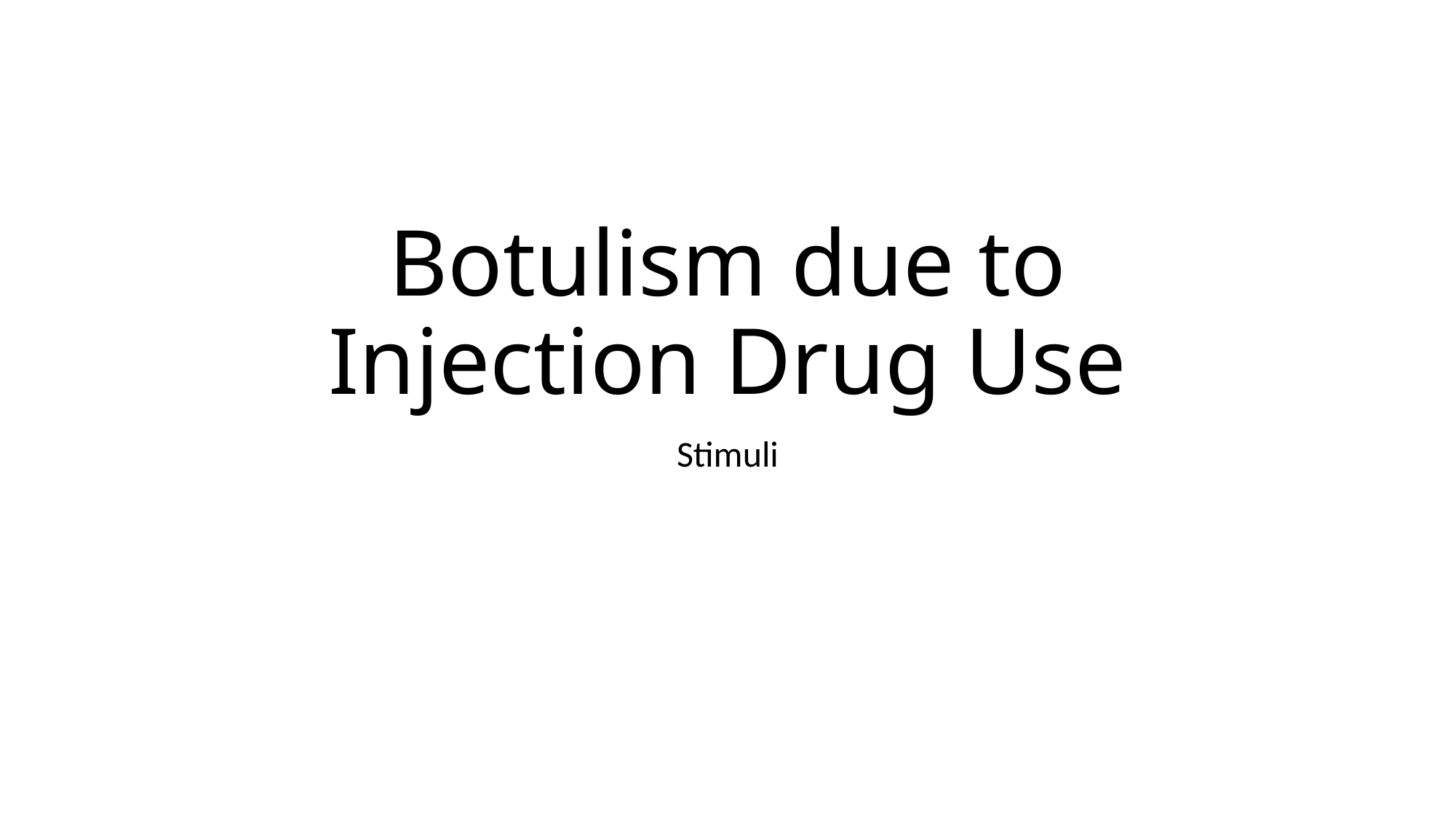

# Botulism due to Injection Drug Use
Stimuli

## Slide 2
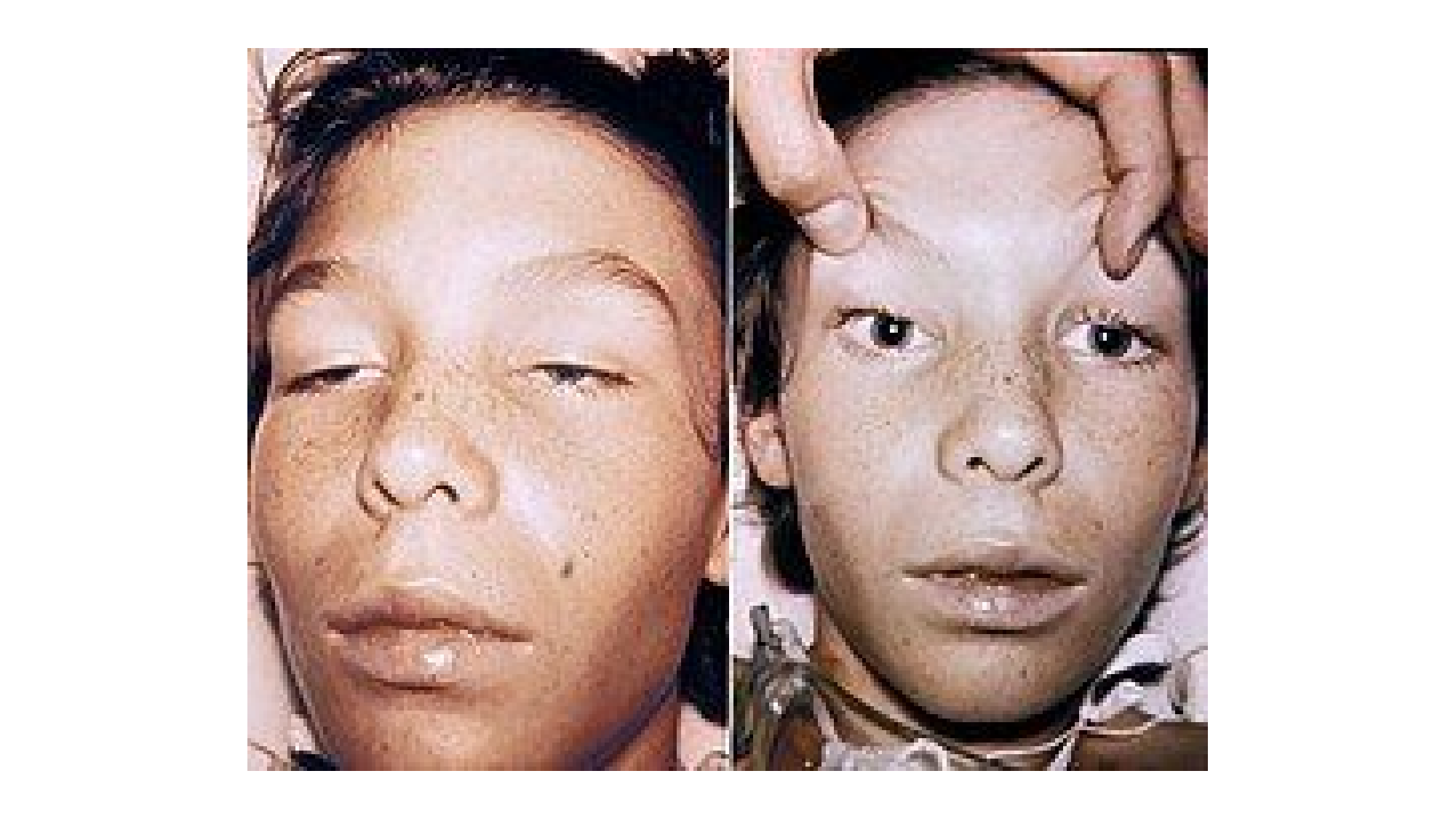

## Slide 3
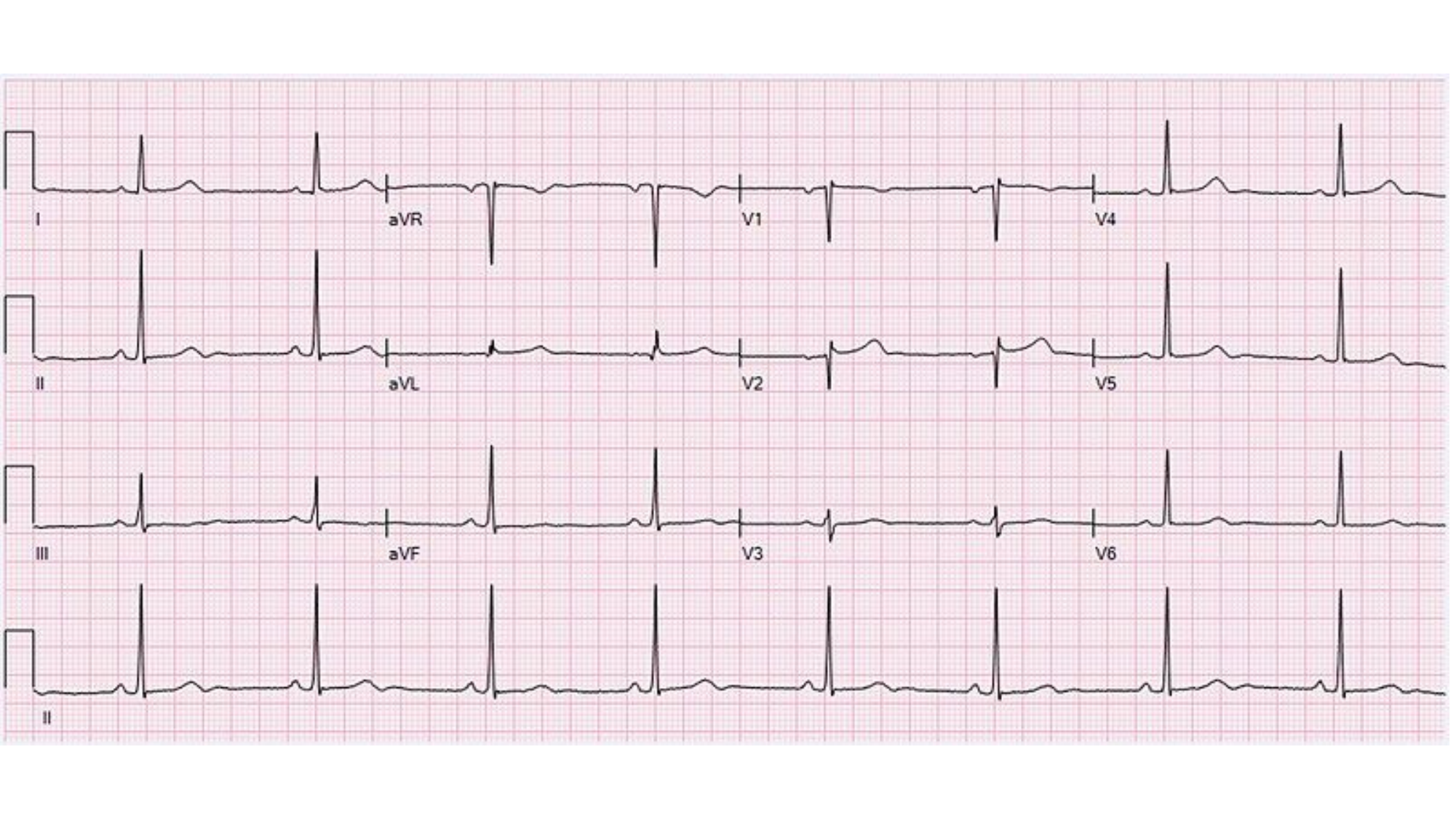

## Slide 4
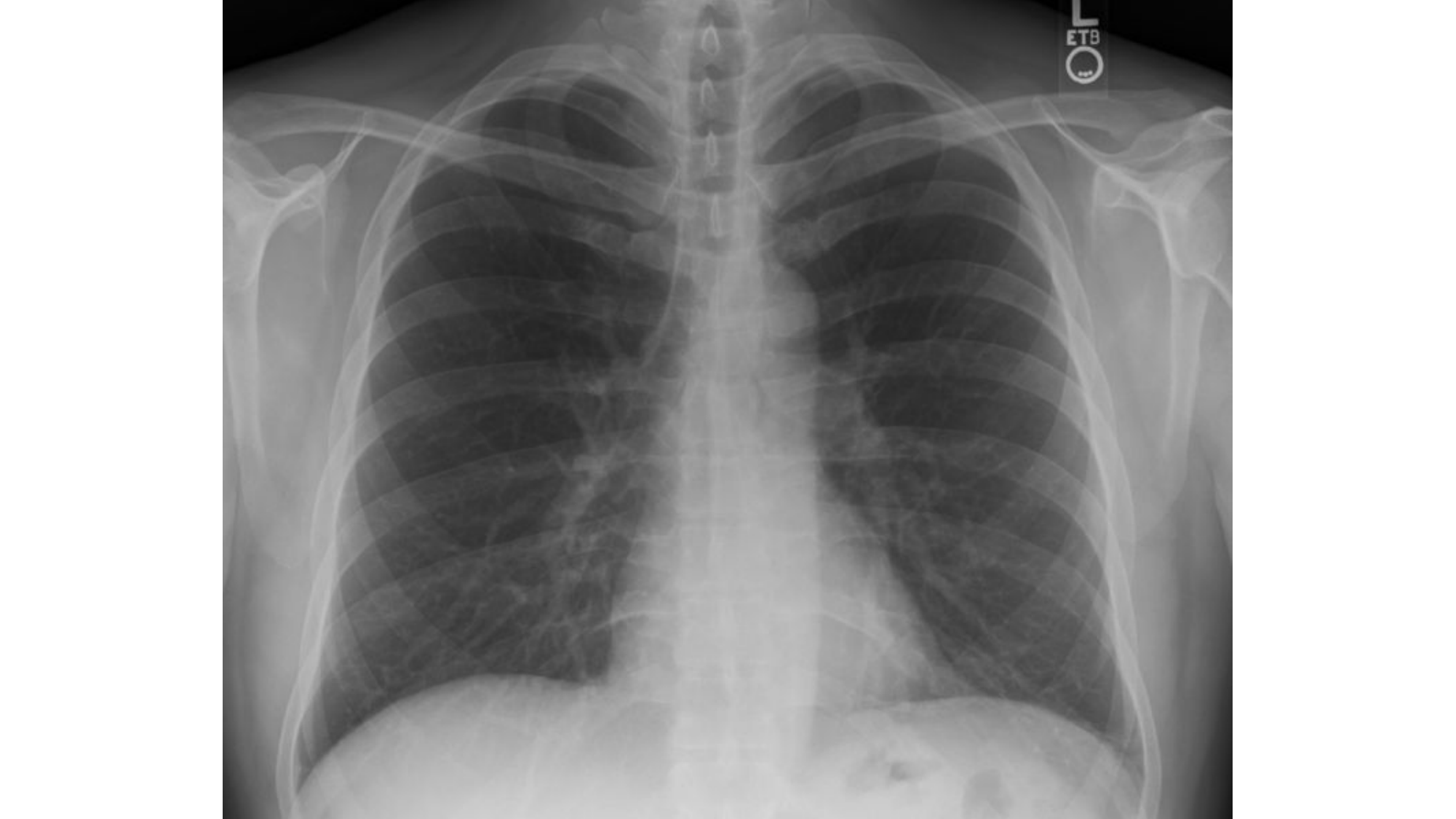

## Slide 5
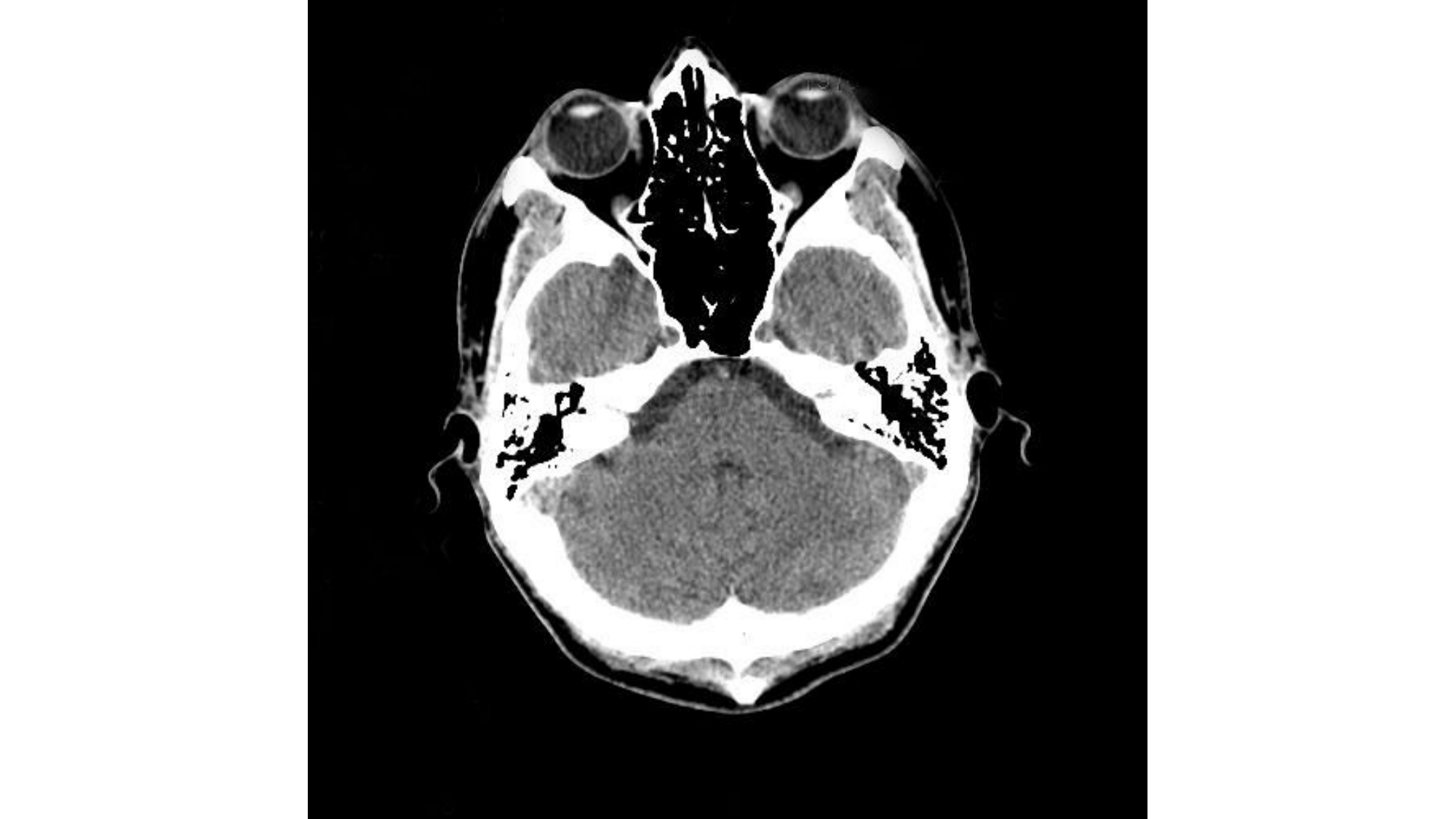

## Slide 6
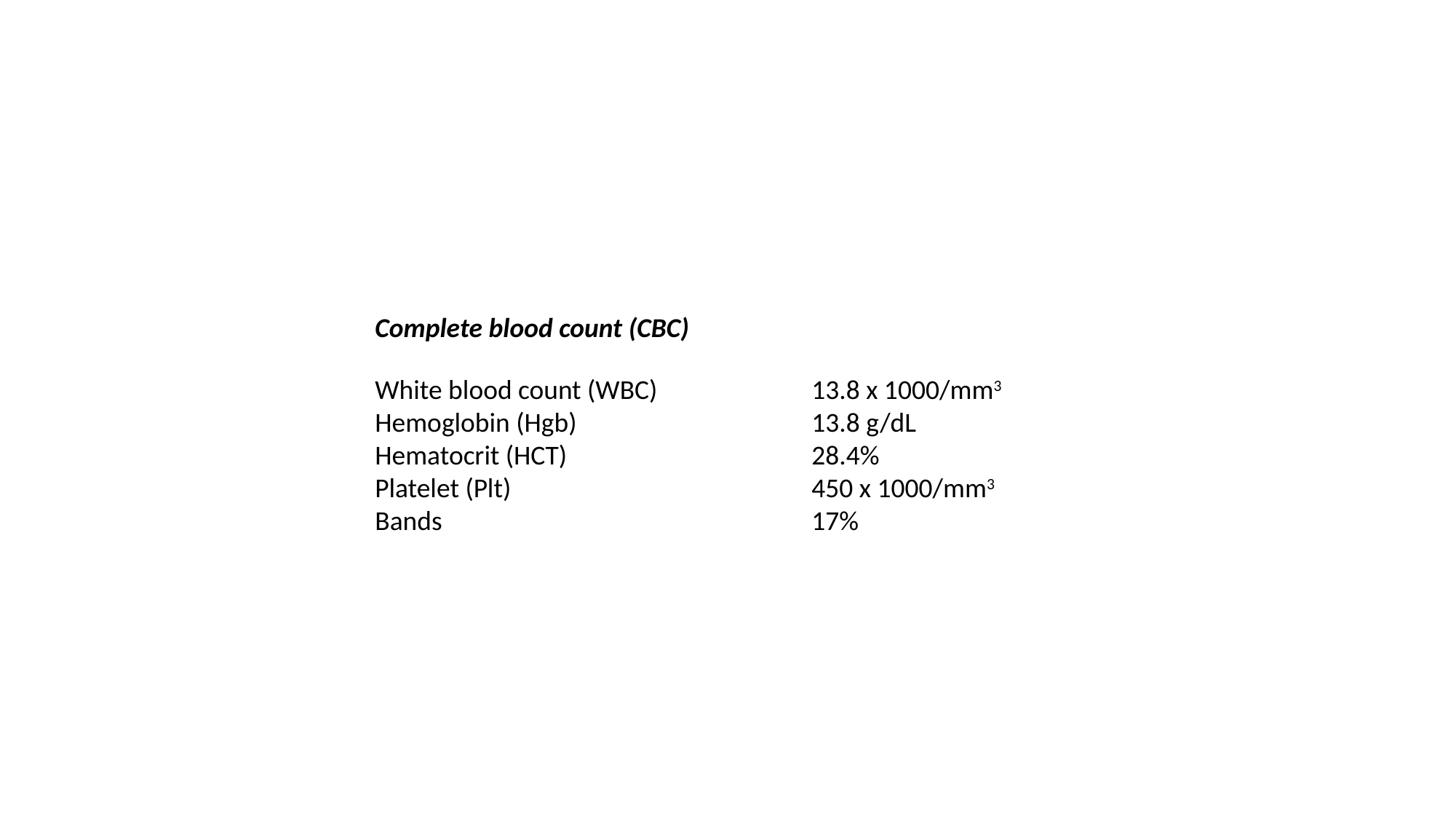

Complete blood count (CBC)
White blood count (WBC)		13.8 x 1000/mm3
Hemoglobin (Hgb)			13.8 g/dL
Hematocrit (HCT)			28.4%
Platelet (Plt)				450 x 1000/mm3
Bands				17%

## Slide 7
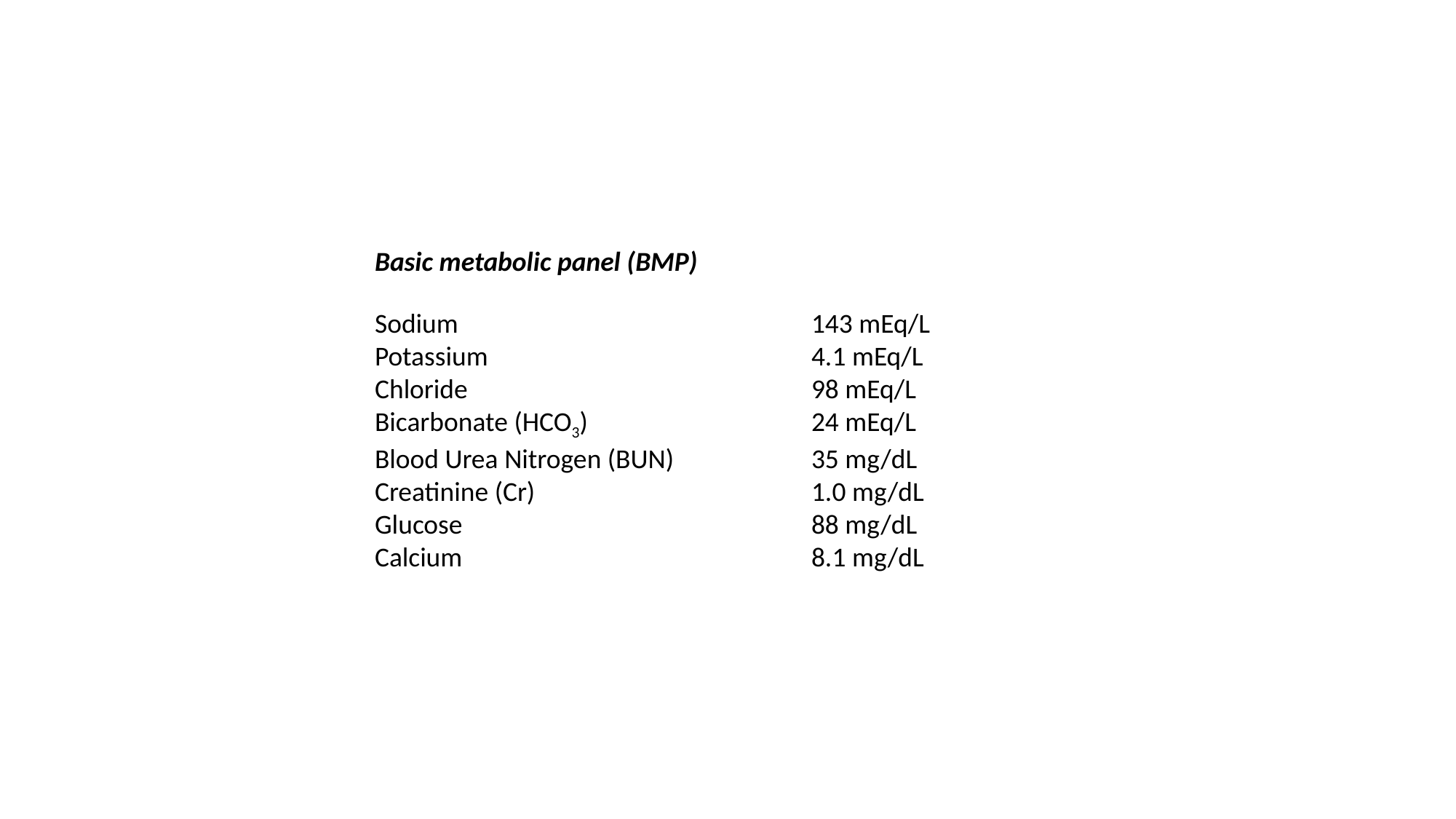

Basic metabolic panel (BMP)
Sodium				143 mEq/L
Potassium				4.1 mEq/L
Chloride				98 mEq/L
Bicarbonate (HCO3)			24 mEq/L
Blood Urea Nitrogen (BUN)		35 mg/dL
Creatinine (Cr)				1.0 mg/dL
Glucose				88 mg/dL
Calcium				8.1 mg/dL

## Slide 8
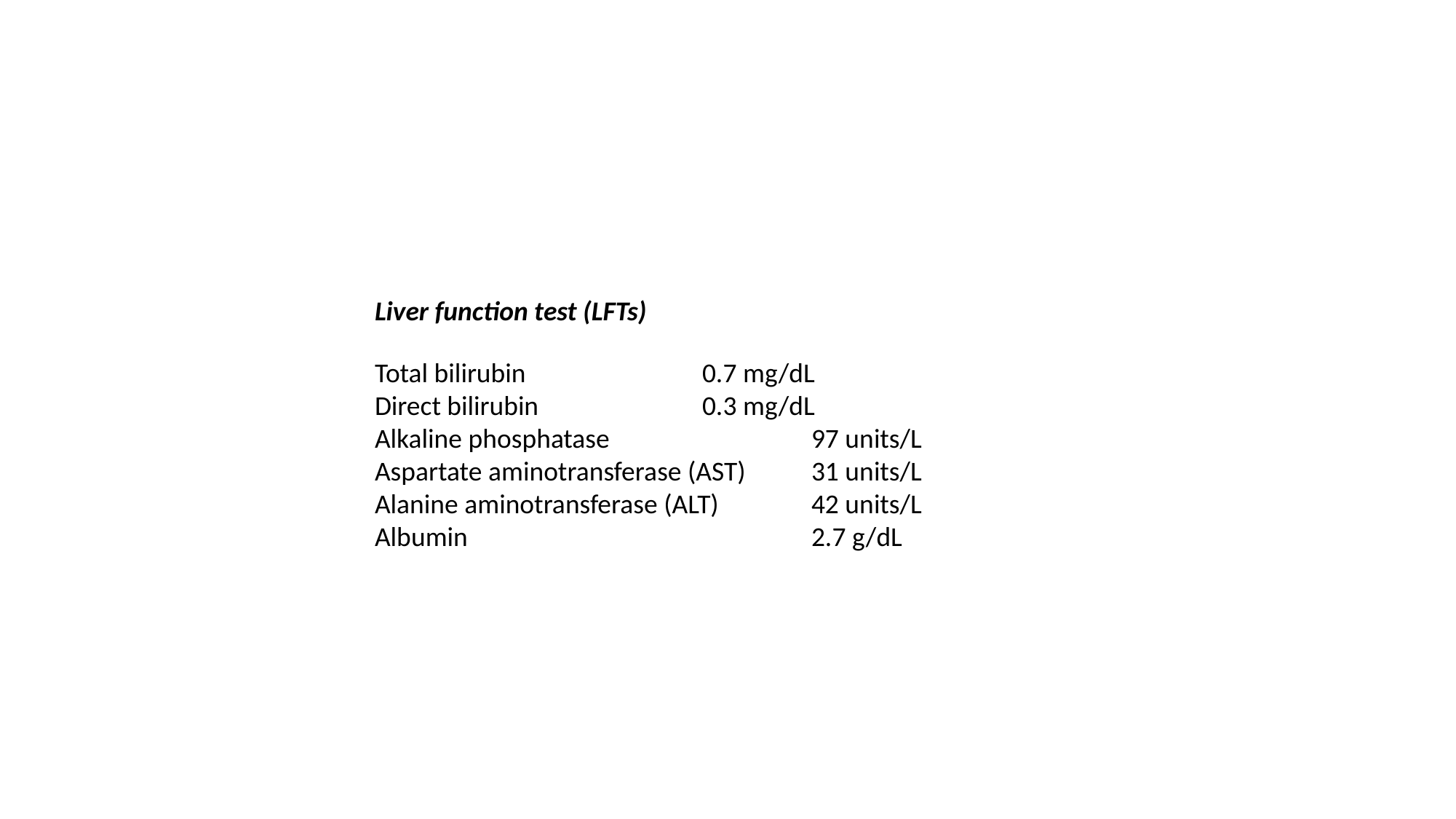

Liver function test (LFTs)
Total bilirubin			0.7 mg/dL
Direct bilirubin			0.3 mg/dL
Alkaline phosphatase		97 units/L
Aspartate aminotransferase (AST)	31 units/L
Alanine aminotransferase (ALT)	42 units/L
Albumin				2.7 g/dL

## Slide 9
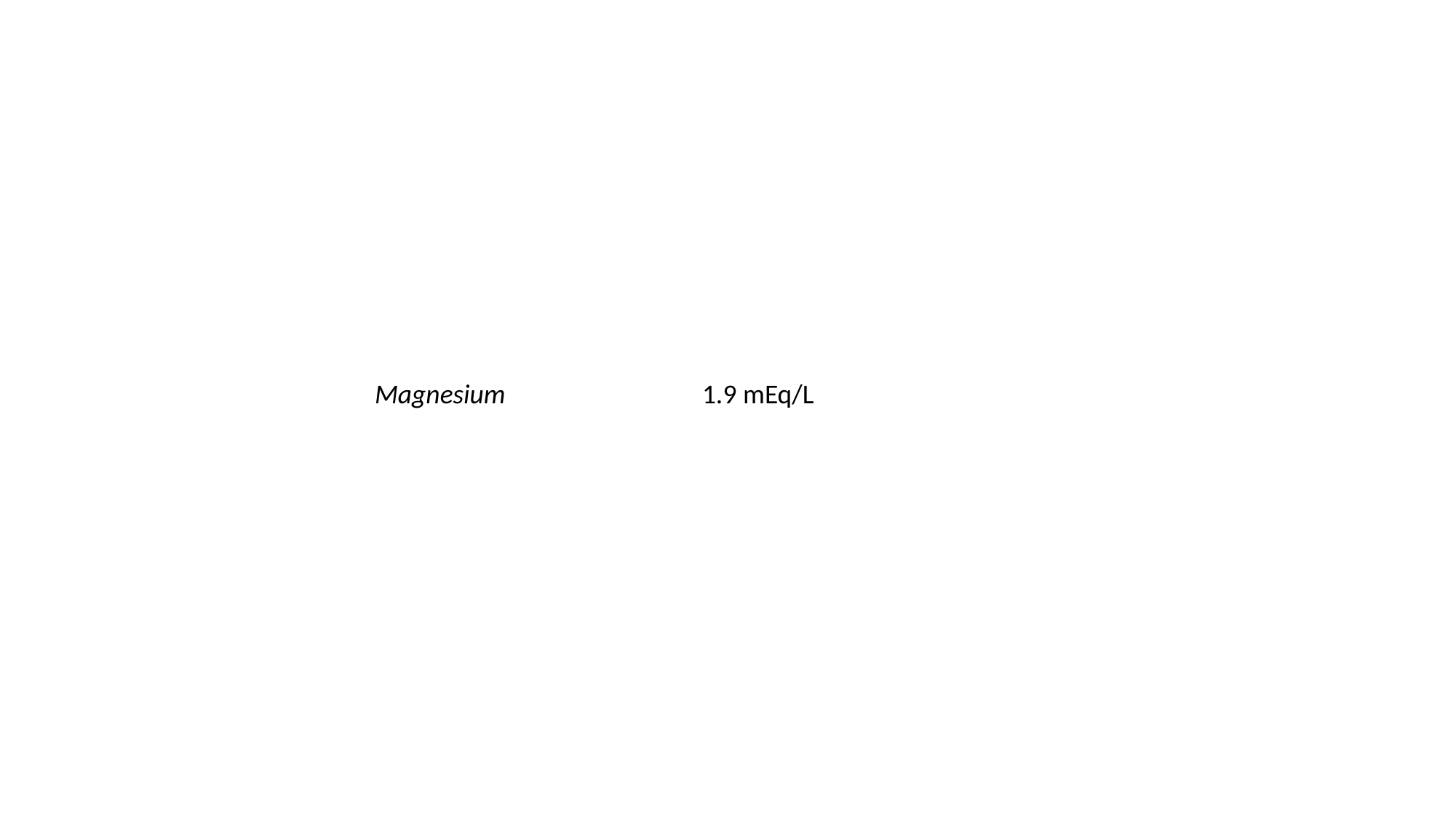

Magnesium		1.9 mEq/L

## Slide 10
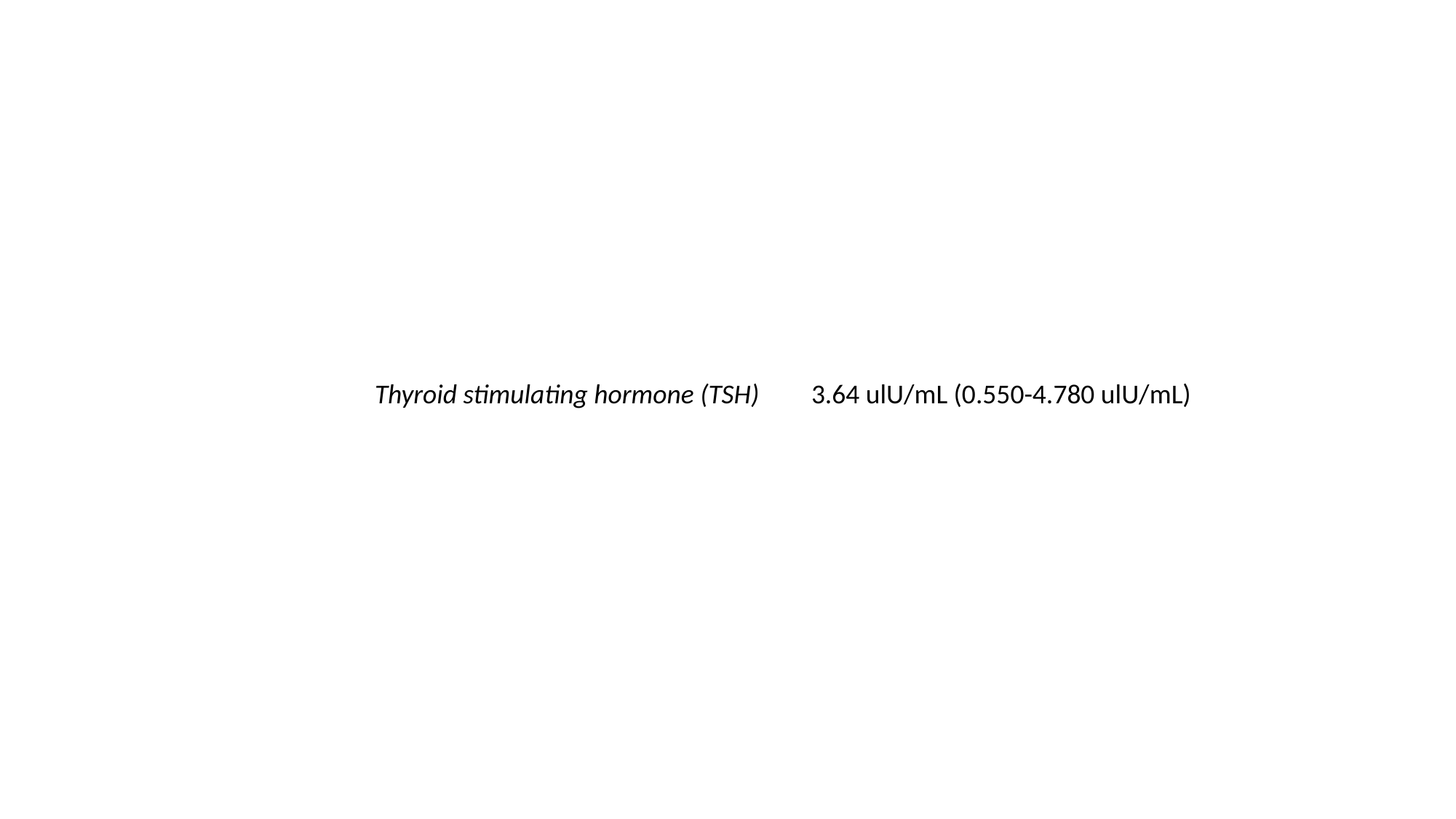

Thyroid stimulating hormone (TSH)	3.64 ulU/mL (0.550-4.780 ulU/mL)

## Slide 11
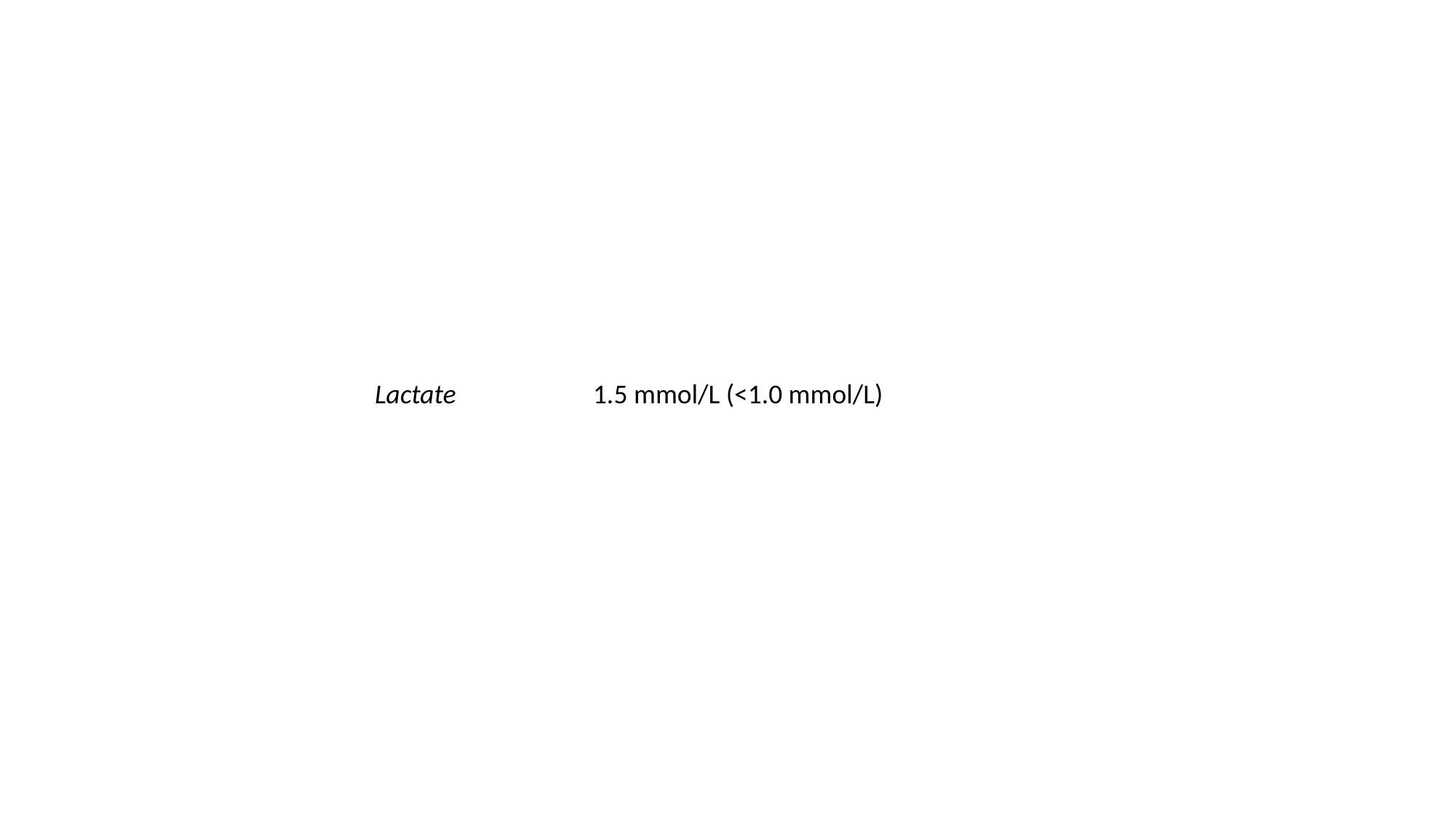

Lactate		1.5 mmol/L (<1.0 mmol/L)

## Slide 12
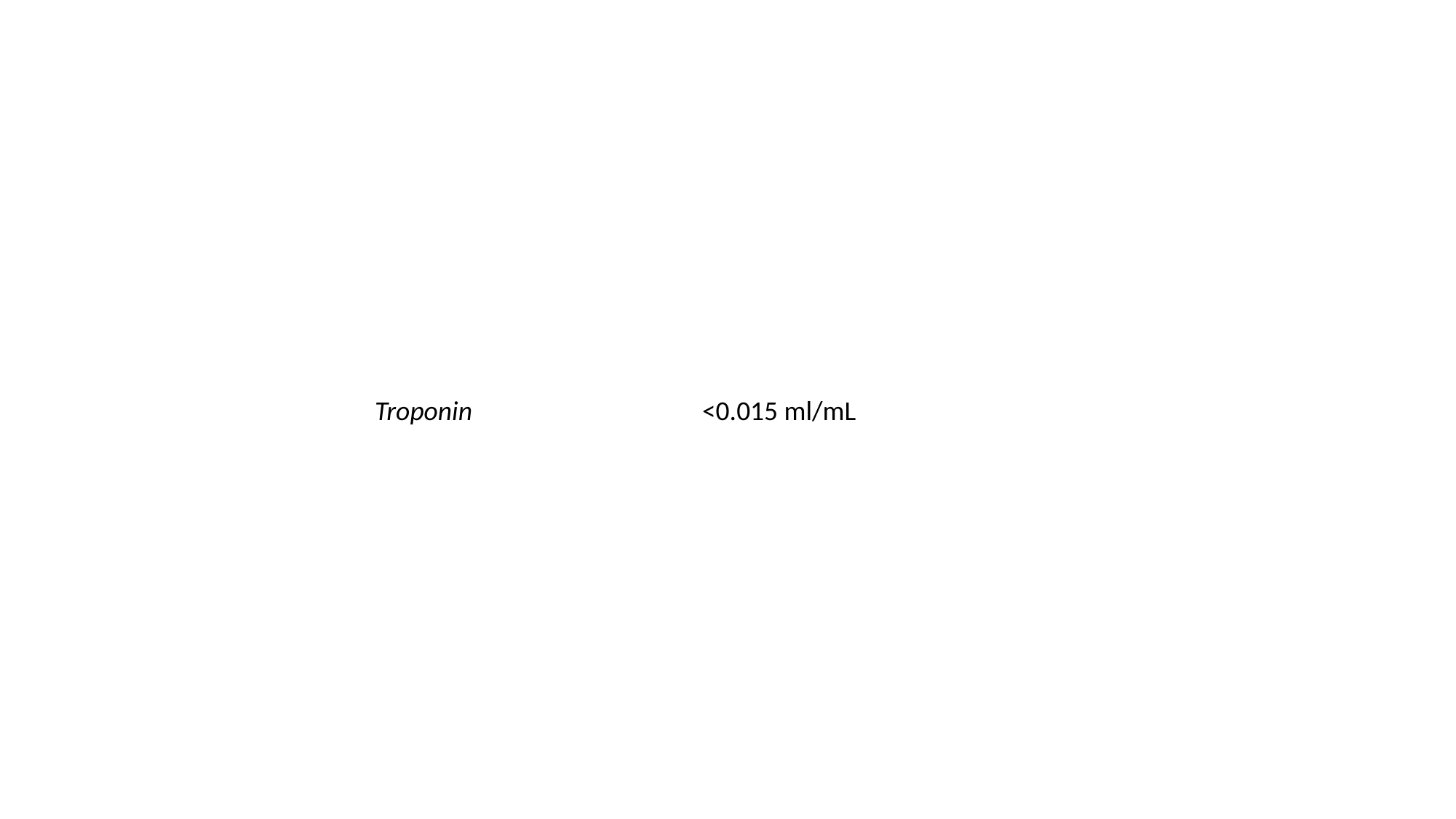

Troponin			<0.015 ml/mL

## Slide 13
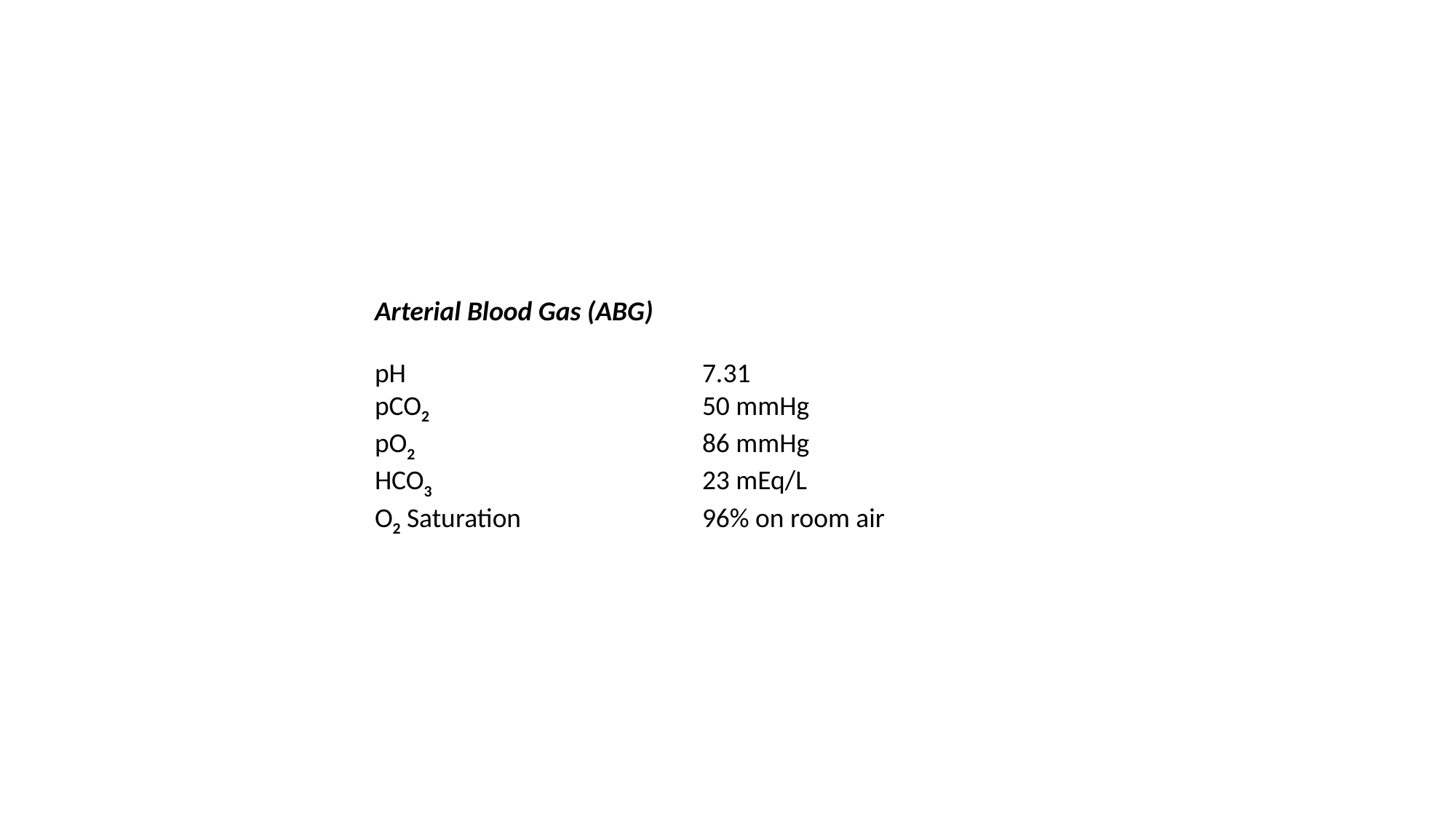

Arterial Blood Gas (ABG)
pH 			7.31
pCO2 			50 mmHg
pO2 			86 mmHg
HCO3 			23 mEq/L
O2 Saturation 		96% on room air

## Slide 14
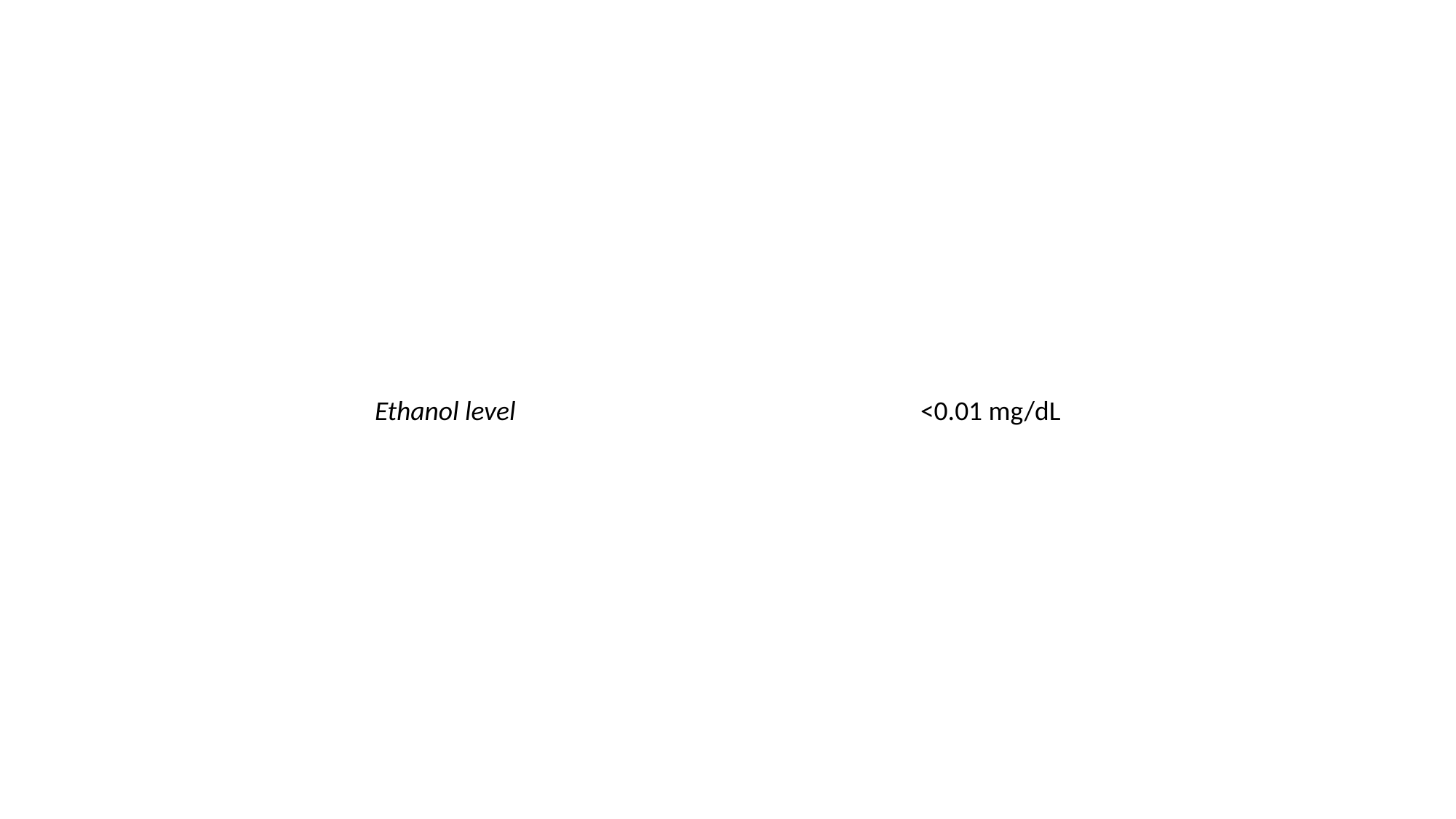

Ethanol level				<0.01 mg/dL

## Slide 15
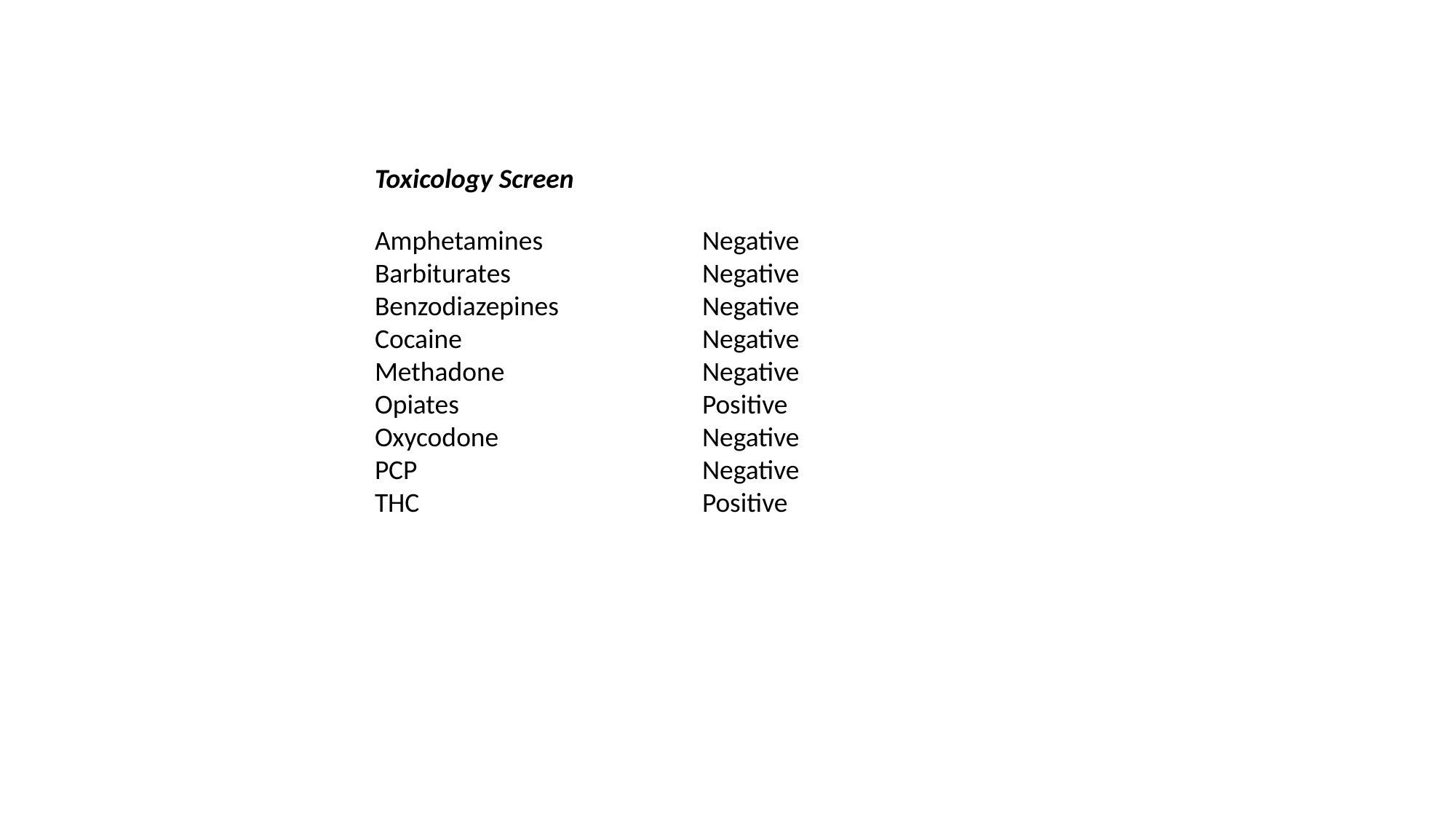

Toxicology Screen
Amphetamines		Negative
Barbiturates		Negative
Benzodiazepines		Negative
Cocaine			Negative
Methadone		Negative
Opiates			Positive
Oxycodone		Negative
PCP			Negative
THC			Positive

## Slide 16
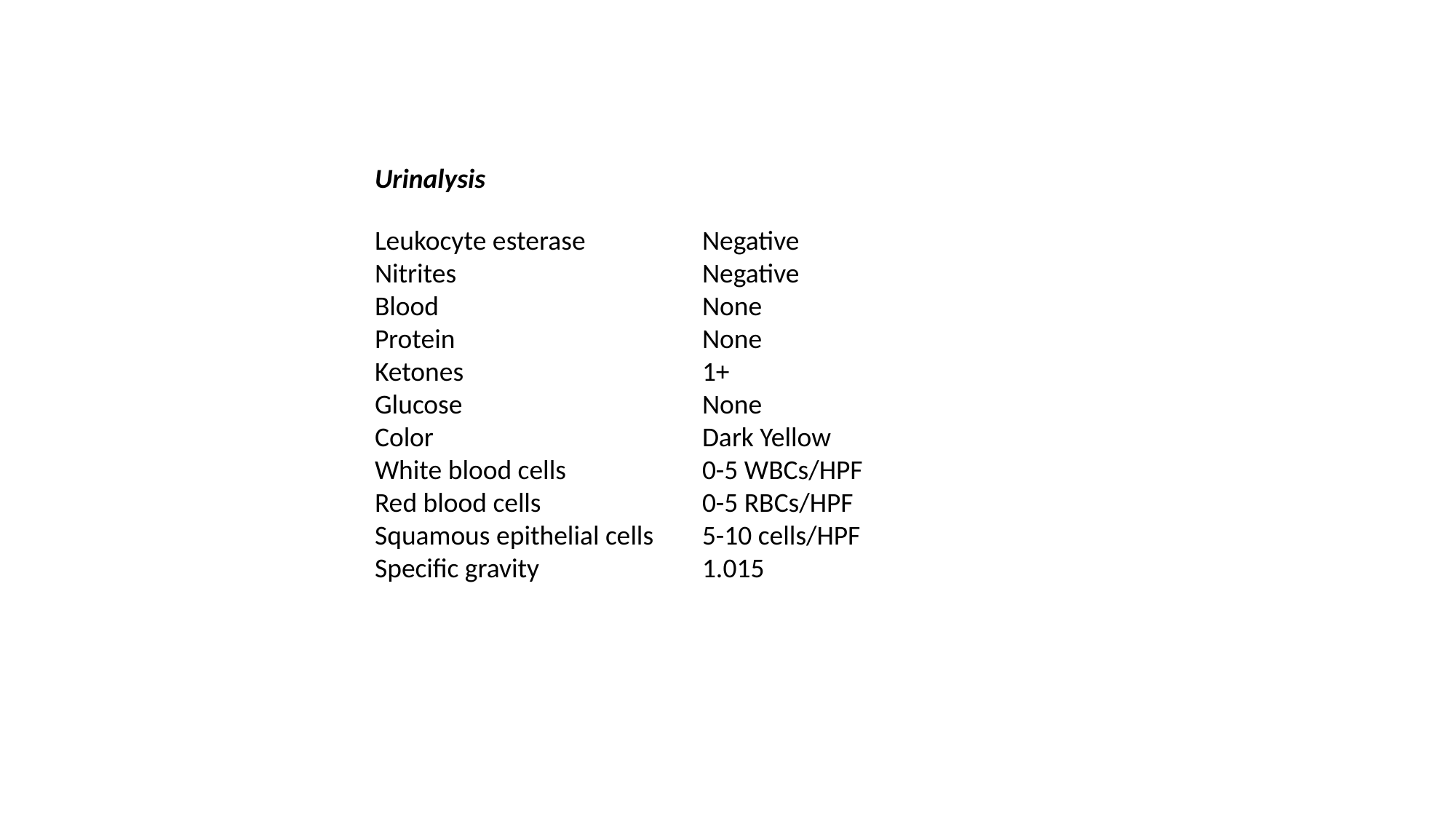

Urinalysis
Leukocyte esterase		Negative
Nitrites			Negative
Blood			None
Protein			None
Ketones			1+
Glucose			None
Color			Dark Yellow
White blood cells		0-5 WBCs/HPF
Red blood cells		0-5 RBCs/HPF
Squamous epithelial cells	5-10 cells/HPF
Specific gravity		1.015

## Slide 17
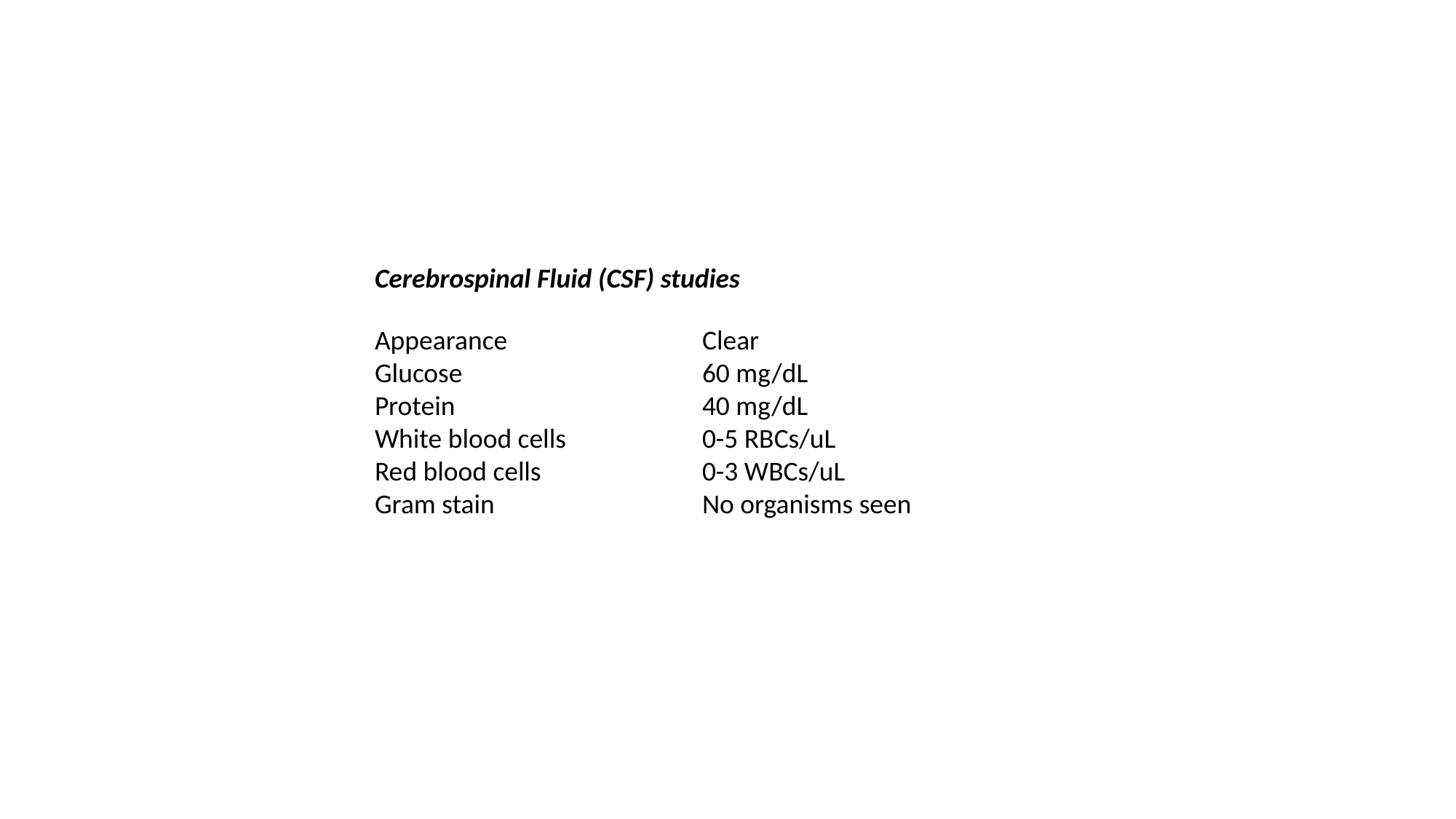

Cerebrospinal Fluid (CSF) studies
Appearance		Clear
Glucose			60 mg/dL
Protein			40 mg/dL
White blood cells 		0-5 RBCs/uL
Red blood cells 		0-3 WBCs/uL
Gram stain		No organisms seen
